# Impact of Change of Length of Admission During COVID-19 for Inpatient Alcohol Detoxification on Relapse to Daily Alcohol Use With 1 Year: A Service Evaluation

**DOI:** 10.1192/bjo.2025.10478

**Published:** 2025-06-20

**Authors:** Aled Davies

**Affiliations:** Swansea Bay University Health Board, Swansea, United Kingdom. Cardiff University, Cardiff, United Kingdom

## Abstract

**Aims:** We reviewed the impact of reducing length of admission during COVID-19 for planned inpatient medically assisted alcohol withdrawal (MAAW) on relapse to daily alcohol use within one year.

We aimed to describe the demographic, social and medical characteristics of patients admitted for a planned MAAW, rate of relapse to alcohol use over time, and identify good aspects of care that improved outcomes.

**Methods:** A retrospective cohort methodology was used using electronic health records. Patients included were identified as alcohol dependent, admitted for a planned inpatient MAAW to a specialist unit within Swansea Bay University Health Board between January 2019 and June 2023.

Patients admitted from March 2020 to April 2022 were identified as the exposed group, and those admitted between January 2019 and February 2020 and May 2022 and June 2023 as the control group.

**Results:** 311 admissions for MAAW were identified (125 in the exposed and 186 in the control group). Demographic and medical characteristics were evenly matched. Mean length of admission in the exposed and control group was 6 and 10 days respectively. 57.2% of admissions had relapsed to daily alcohol use by 52 weeks, comparable with existing research.

Time-to-event analysis identified the median time to relapse as 22 weeks and 26 weeks in exposed and control groups respectively.

Hazard ratio of 1.20 (95% confidence interval 0.89–1.61, p-value 0.22) was found in the risk of relapse in the exposed group compared with the control group, suggesting a 20% higher risk of relapse in the exposed group compared with the control by 52 weeks. However, this was not statistically significant.

The hazard ratio for relapsing if discharged on relapse prevention medication (RPM) was 0.50 (95% CI 0.31–0.78, P-value 0.002), suggesting a 50% benefit to remaining abstinent at 52 weeks if discharged on RPM. Similarly, prescribing disulfiram after MAAW, had a hazard ratio of 0.39 (95% CI 0.26–0.58, P-value 0.000004), reducing the risk of relapse by 61%.

**Conclusion:** We were able to characterise the demographic and medical background of patients receiving planned inpatient MAAW, which will help in future design and delivery of specialist MAAW units. No evidence was found to support a reduction in the length of admission for an inpatient MAAW. RPM significantly reduced the risk of relapse, especially the use of disulfiram. Several combinations of RPMs were prescribed, highlighting the need to standardise prescribing of RPMs post MAAW.

